# Supplementary material for: Exploring the Potential Mechanism of Prothioconazole Resistance in Fusarium graminearum in China
Source: J Fungi (Basel). 2023 Oct 10;9(10):1001. doi: 10.3390/jof9101001 (PMC10607755; doi:10.3390/jof9101001)
Supplement: Supplementary file 1 [file jof-09-01001-s001.zip › jof-2627892-supplementary.pdf]

Supplementary Table S1 Fungicides used in this study

**Fungicides**	**Active ingredient concentration (%)**	**Manufacturers**
Prothioconazole	97.0	Kangbaotai Fine-Chemical Co. Ltd.
Tebuconazole	96.2	Sheyang Huanghai Pesticide Chemical Co. Ltd.
Prochloraz	95.0	Kangbaotai Fine-Chemical Co. Ltd.
Carbendazim	98.1	Haili Guixi Chemical Co., Ltd.
Pyraclostrobin	97.5	Kangbaotai Fine-Chemical Co. Ltd.
Fluazinam	96.0	Hubei Jianyuan Chemical Co. Ltd.

Supplementary Table S2 Primers used in this study

**Primers**	**Sequences（5′-3′）**	**Relevant characterstics**
FgCYP51A-F	ATGTTCCATCTACTCATCTATC	Amplification of the FgCYP51A gene
FgCYP51A-R	CTATATCTTCTTCCTACGCTCC	
FgCYP51B-F	ATGGGTCTCCTTCAAGAAC	Amplification of the FgCYP51B gene
FgCYP51B-R	TTACTGGCGTCGCTCCCAGTG	
FgCYP51C-F	ATGGAATCGCTCTACGAGAC	Amplification of the FgCYP51C gene
FgCYP51C-R	TCATTCTACTGTCTCGCGTC	
Rt -Fg-actin-F	GTCCACCTTCCAGCAAATGT	Reference gene relative expression level
Rt -Fg-actin-R	CCCAAAGCTTAGCGTCTGTC	
RT-FgCYP51A-F	AGCCCGTACTTGCCCTTTGG	Analyzing the FgCYP51A gene relative expression level
RT-FgCYP51A-R	GGGCGGGTCGTGAGAACAAA	
RT-FgCYP51B-F	GAGTCCCTGGCCGCTCTCTA	Analyzing the FgCYP51B gene relative expression level
RT-FgCYP51B-R	GCGGCGCTCCTTGATAGTGT	
RT-FgCYP51C-F	TTCGTCTCCCGGCACAATGG	Analyzing the FgCYP51C gene relative expression level
RT-FgCYP51C-R	CGTCCAGCTCCAAAGGGCAA	
